# Subsets of Memory CD4^+^ T Cell and Bactericidal Antibody Response to *Neisseria meningitidis* Serogroup C after Immunization of HIV-Infected Children and Adolescents

**DOI:** 10.1371/journal.pone.0115887

**Published:** 2014-12-22

**Authors:** Lucimar G. Milagres, Priscilla R. Costa, Giselle P. Silva, Karina I. Carvalho, Wânia F. Pereira-Manfro, Bianca Ferreira, Daniella M. Barreto, Ana Cristina C. Frota, Cristina B. Hofer, Esper G. Kallas

**Affiliations:** 1 State University of Rio de Janeiro, Department of Microbiology, Immunology and Parasitology, Rio de Janeiro, RJ, Brazil; 2 Division of Clinical Immunology and Allergy, School of Medicine, University of São Paulo, São Paulo, SP, Brazil; 3 Instituto de Puericultura e Pediatria Martagão Gesteira – Federal University of Rio de Janeiro, Rio de Janeiro, RJ, Brazil; 4 Preventive Medicine Department, School of Medicine – Federal University of Rio de Janeiro, Rio de Janeiro, RJ, Brazil; Federal University of São Paulo, Brazil

## Abstract

Meningococcal disease is endemic in Brazil, with periodic outbreaks and case fatality rates reach as high as 18 to 20% of cases. Conjugate vaccines against meningococci are immunogenic in healthy children. However, we have previously shown a poor bactericidal antibody response to a Men C conjugate vaccine in Brazilian HIV-infected children and adolescents after a single vaccine administration. The goal of the present work was to investigate associations between bactericidal antibody response induced by MenC vaccine and the frequency and activation profile (expression of CD38, HLA-DR and CCR5 molecules) of total CD4^+^ memory T cell sub-populations in HIV-1-infected children and adolescents. Responders to vaccination against MenC had a predominance (about 44%) of CD4^+^ T_INTERMEDIATE_ subset followed by T_TRANSITIONAL_ memory subset (23 to 26%). Importantly, CD4^+^ T_INT_ frequency was positively associated with bactericidal antibody response induced by vaccination. The positive correlation persisted despite the observation that the frequency T_INT_ CD38^+^HLA-DR^+^ was higher in responders. In contrast, CD4^+^ T_CENTRAL MEMORY_ (T_CM_) subset negatively correlated with bactericidal antibodies. In conclusion, these data indicate that less differentiated CD^+^ T cells, like T_CM_ may be constantly differentiating into intermediate and later differentiated CD4^+^ T cell subsets. These include CD4 T_INT_ subset which showed a positive association with bactericidal antibodies.

## Introduction

The development of immune memory mediated by T lymphocytes is central to durable, long-lasting protective immunity. A key issue is how to direct the generation and persistence of memory T cells and to elicit the effective secondary responses to protect against a given pathogen [1], [2]. This is particularly important in the setting of people living with HIV, where CD4^+^ T cells are the main target of viral replication and suffer from bystander activation [3], [4].

Meningococcal disease (MD) is endemic in Brazil, with periodic outbreaks [5] and an incidence rate of 1.4–2.5 cases per 100,000 inhabitants [5]. Case fatality rates reach as high as 18 to 20% of cases [5], [6]. Since 2000, Brazil has experienced an increase in serogroup C MD. In 2013, MD accounted for 70% of reported cases to the Brazilian Ministry of Health [6]. In 2006, the Brazilian National Immunization Program suggested that one dose of the conjugate vaccine against *N. meningitidis* serogroup C (MenC) should be given to all HIV-infected children aged 2 to 13 years-old [7].

Conjugate vaccines against meningococci are immunogenic in healthy children [8]. The majority of available immunogenicity studies have demonstrated the induction of antigen-specific memory cells indirectly through the measurement of recall antibody response to a booster dose of vaccine administered long after the primary vaccine series [8]. We have previously shown a poor bactericidal antibody response to a Men C conjugate vaccine in Brazilian HIV-infected children and adolescents after a single vaccine administration [9]. In a second study [10], we demonstrated that pre-existing higher CD4^+^ T cell activation leads to poor MenC vaccine response in children living with HIV.

Memory CD4^+^ and CD8^+^ T cells have distinct phenotypes and differentiation status [11], [12]. Flow cytometry T cell phenotyping allows the identification of five subsets of memory cells: T central memory (T_CM_), T transitional memory (T_TM_), T intermediary memory (T_INT_), T effector memory (T_EM_) and T effector cells (T_Eff_) based on CD45RA, CCR7 and CD27 proteins expression [11], [12]. Burgers et al [11] ranked the CD8^+^ T cell memory subpopulations based on the predicted ability to survive and proliferate from highest to lowest: T_Naive_ →T_CM_ →T_TM_ → T_INT_→ T_EM_ → T_Eff_. However, this lineage differentiation is not fixed, specially for CD4^+^ T cells which show a inherent plasticity [2]. Immune hyperactivation, skewed T-cell differentiation, senescence, exhaustion, anergy and loss of functionality are hallmarks of progressive HIV-1 infection [13], [14].

The goal of the present work was to investigate associations between bactericidal antibody response induced by MenC vaccine and the frequency and activation profile of total CD4^+^ memory T cell sub-populations in HIV-1-infected children and adolescents.

## Materials and Methods

### Ethics statement

This study was approved by the *Instituto de Puericultura e Pediatria Martagão Gesteira, Universidade Federal do Rio de Janeiro* (IPPMG/UFRJ), Institutional Review Board (IRB, number 24/09) and Brazilian Ministry of Health Ethics Comission (*Comissão Nacional de Ética em Pesquisa*, CONEP, number 15578).

### Study design and population

We conducted a prospective cohort study at the *Instituto de Puericultura e Pediatria Martagão Gesteira, Universidade Federal do Rio de Janeiro* (IPPMG/UFRJ), Rio de Janeiro, Brazil, to investigate the secoronversion rate after MenC vaccination in HIV-vertically infected 2–18 year-old children. Participants were enrolled between January 2011 and December 2012, meeting the following eligibility criteria: evidence of HIV infection at the moment of the study enrollment; CD4^+^T cell count ≥350 cells/µl or ≥15%; no evidence of other cause for severe immune suppression; and no antibiotic use within 2 weeks prior to immunization. With one exception (one individual who responded to the vaccine), all individuals were receiving HAART (defined as three different antiretrovirals, from at least two different drug classes) for more than 3 months. All participant's parents or legal guardians provided written informed consent, as well as the participants who were aware of their HIV-infection status.

### Study protocol

For all participants who met the inclusion and exclusion criteria, the research physician at IPPMG HIV/AIDS Pediatric Clinic approached the patient and their parent or legal guardian offering to participate in this study. After voluntary acceptance and signature of the IRB-approved Informed Consent, the study team checked the eligibility criteria, collected baseline clinical samples and administered an intramuscular injection of MenC vaccine (Novartis; C Polysaccharide/CRM_197_) at the recommended dose (10 µg/0.5 ml). Blood samples were collected before (Visit 1) and 1 to 2 months after immunization (Visit 2). Heparin-treated tubes or in the absence of anti-coagulant were used and processed within 3 hours after the blood draw. Peripheral blood mononuclear cells (PBMC) were separated by density-gradient centrifugation over Histopaque (Sigma, St Louis, USA) and stored in RPMI/20% fetal bovine serum/10% DMSO in liquid nitrogen until the day of the assays. Serum samples were stored at −70°C or −20°C until the day of anti-MenC antibody titers measurements.

### Bactericidal assay

Serum bactericidal antibody titers were measured as previously described [15]. Briefly, the final reaction mixture contained 25 µl of diluted test serum, previously heat inactivated at 56°C for 30 min, 12.5 µl of human serum without detectable intrinsic bactericidal activity as a complement source, and 12.5 µl of log phase meningococci (about 5×10^3^ CFU/ml). The bactericidal reaction was carried out at 37°C for 60 min. The bactericidal titer was defined as the reciprocal of the serum dilution causing ≥50% killing.

In this study, seroconversion was defined as a≥4-fold increase in serum bactericidal antibody titers after vaccination. This criterion was utilized to define the two groups of participants: those with documented seroconversion (Sc+) and those without seroconversion (Sc-). We randomly selected 18 participants who responded (Sc+) to the vaccine and 18 who did not respond (Sc-) after immunization against MenC.

Two local strains of MenC were used as target strains: N79/96 (C:2b:P1.10) and N753/00 (C:23:P1.14-6), both kindly provided by Adolfo Lutz Institute, Bacteriology Section, São Paulo, SP, Brazil.

### Flow Cytometry

Flow cytometry assays were performed at the *Laboratório de Investigação Médica 60*, Division of Clinical Immunology and Allergy, School of Medicine, University of São Paulo, as previously described [10].

**Figure 1:**
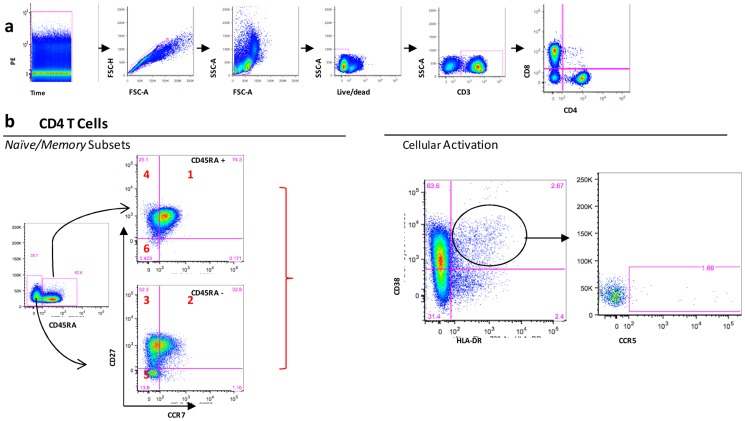
Strategy of analysis of Flow Cytometer data from one representative experiment with PBMC samples of HIV infected children. (A) Were used high Quality Parameters: (Time x PE-to check laser status; Singlets-to exclude the doublets cells; SSC x FSC- to choose lymphocytes subset; Live/Dead-to exclude the dead cells; gate SSC x CD3- to chose T lymphocytes and gate CD4 x CD8- to separate in CD4 and CD8 T subsets, then b Naïve/Memory subsets and cellular activation were evaluated in CD4 T Cells and CD8 T Cells, (B) Naïve/Memory CD4^+^ Subsets were classified through differentiated expression of three markers (CD45RA, CD27 and CCR7): 1- Naïve (CD45RA^+^CD27^+^CCR7^+^); 2- Central Memory (CD45RA^−^CD27^+^CCR7^+^); 3- Transitory Memory (CD45RA^−^CD27^+^CCR7^−^); 4- Intermediary Memory (CD45RA^+^CD27^+^CCR7^−^); 5- Effector Memory (CD45RA^−^CD27^−^CCR7^−^); 6- Effector (CD45RA^+^CD27^−^CCR7^−^); Cellular Activation were characterized through expression of CD38, HLA-DR and CCR5.

Briefly, frozen PBMCs were quickly thawed in a 37°C water bath, cells were counted, and 10^6^ cells were stained with phycoerythrin (PE)-Texas Red-conjugated CD3 (clone UCHT1); pacific blue-conjugated CD4 (clone RPA-T4), PE-conjugated CD27 (clone L128), FITC-conjugated CD45RA (clone L48), PE-Cy7-conjugated CCR7 (clone 3D12), PerCP-Cy5.5-conjugated CD38 (clone HIT2); APC-conjugated CCR5 (clone 2D7/CCR5); Alexa 700-conjugated HLA-DR (clone G46-6). For all mAbs, fluorescence-minus-one (FMO) controls were used to determine positive and negative boundaries. Fluorescence compensation was calculated with the signals from fluorochrome monoclonal antibodies linked to CompBeads (BD). Samples were run in a FACSFortessa flow cytometer (BD Bioscences) and data were stored using Diva Software for further analyses. Fig. 1 shows representative dot plots with the strategies of analyses used to gate the different T cell populations described in this study.

### Statistical analysis

Flow cytometry graphs were generated using FlowJo software, version 7.6.4 (Tree Star Inc., Ashland, OR). Statistical analyses were performed using STATA program, version 9.0 (Texas, USA). Data were expressed as median values and statistical analysis of significance was calculated using non-parametric Kruskal-Wallis test. All tests were two-tailed, and a P<0.05 was considered as significant. The correlation between different measurements of immune response was analyzed using Spearman rank test, after graph analyses.

## Results

Previously published data [10] showed no significant differences in age, length of HAART, CDC clinical category and viral load between Sc+ and Sc- groups. Despite a higher nadir and current CD4^+^ T-cell counts in responders, the differences did not reach significant levels. There were no significant correlations between bactericidal titer and age, *nadir* CD4^+^ T-cell count/percentage or CD4^+^ T-cell count/percentage or viral load at the time of vaccination.

### CD4^+^ Intermediate memory T-cell predominates in responders

**Figure 2:**
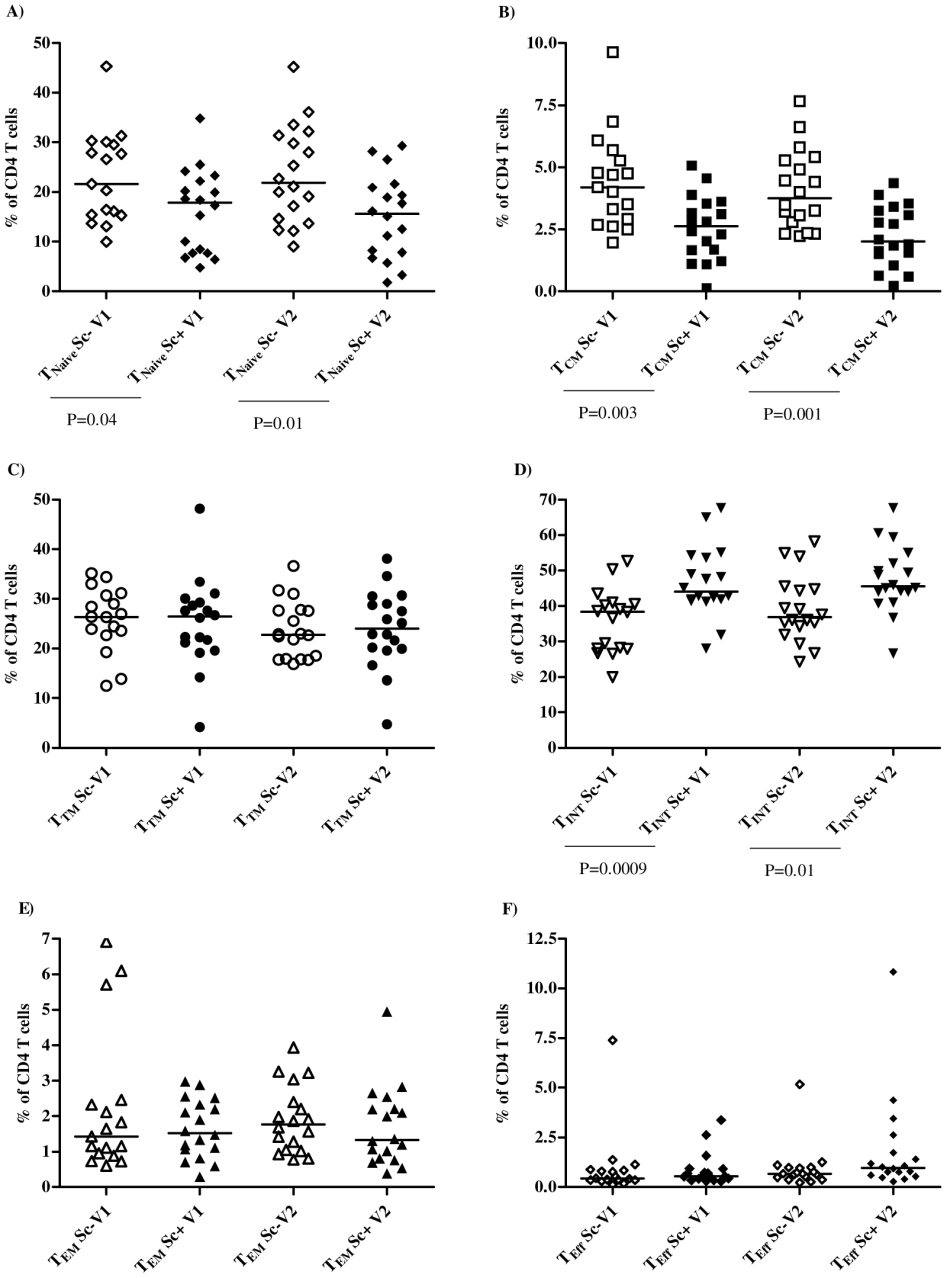
Predominance of CD4^+^ T_INT_ memory subset among HIV^+^ seroconverters. Frequency and median (lines) of CD4^+^ T_Naive_ (A), T_CM_ (B), T_TM_ (C), T_INT_ (D), T_EM_(E) and T_Eff_ (F) subpopulations in seroconverters (Sc+, closed symbols) and non-seroconverters (Sc-, open symbols) before (V1) and after (V2) vaccination.

Fig. 2 shows the proportion of six total CD4^+^ T cell subpopulations in PBMC collected prior (V1) and 1 to 2 months after vaccination (V2) from individuals who seroconverted (Sc+) or not (Sc-) after vaccination against MenC. There were no significant differences between V1 and V2 for all T cell subsets analysed and represented in Fig. 2. Below we describe the median and p-values for V1 samples.

As observed in Fig. 2, T_INT_ subset (Fig. 2D) showed a greater proportion (P<0.01) in both Sc+ and Sc- individuals than the other five CD4^+^ T cell subsets. The frequency of T_INT_ was higher in Sc+ (median of 44%) than in Sc- group (median of 38%, P<0.01). In contrast, Sc+ group had less T_Naive_ cells (median of 18%, P = 0.04, Fig. 2A) than the Sc- group (median of 22%). Of note, T_CM_ levels (Fig. 2B) were significantly smaller in Sc+ group (median of 2.6%) compared with the Sc- group (median of 4.2%, P<0.01). After T_INT_, T_TM_ (Fig. 2C) was the second in frequency (median varying from 23% to 26%) among the six CD4^+^ T cell subsets, but did not differ between the two vaccinee groups. Finally, the effector arms of CD4^+^ T cells, T_EM_ and T_Eff_ cells (Fig. 2E and 2F, respectively) presented a median of ∼1.5% and ∼0.7%, respectively, without significant differences between groups. Therefore, these data indicate contrasting frequency of T_INT_ (higher) and T_CM_ (lower) CD4^+^ T cell subpopulations in individuals who seroconverted compared to those who did not.

### Immune activation levels of CD4^+^ T cell subsets

We next evaluated the frequency of expression of molecules associated with CD4^+^ T cells activation; CD38, HLA-DR (hereafter described as DR) and CCR5 (Table 1). In the Sc- group, T_TM_ was the subset of CD4^+^ T memory cells with the highest (P<0.01) expression of CD38 and DR (median of 0.79%) followed by T_INT_ (median of 0.49%) and T_EM_ (median of 0.42%). For Sc+ individuals we observed a similar profile as described for Sc- except that the activation of T_TM_ (median 0.98%) and T_INT_ (median of 0.78%) were statistically similar due to a higher (P = 0.02) frequency of activated T_INT_ subset compared with Sc-. In both groups, T_CM_ was the less activated CD4^+^ T cell subset (median of 0.07%).

**Table 1:** Frequency (median) of CD4^+^ T cell subsets expressing different combinations of CD38, HLA-DR and CCR5 molecules in PBMC collected before vaccination (V1).

CD4^+^ T cell subsets	CD38^+^DR^+^		CD38^+^DR^+^CCR5^+^		CD38^−^DR^−^CCR5^+^	
	Sc−	Sc+	Sc−	Sc+	Sc−	Sc+
T_Naive_	0.23	0.20	0.009*	0.006	0.003	0.002
T_CM_	0.07	0.07	0.002	0.001	0.004*	0.001
T_TM_	0.79	0.90	0.020	0.015	0.112*	0.047
T_INT_	0.49	0.78*	0.015	0.018	0.016*	0.011
T_EM_	0.42	0.43	0.018*	0.010	0.062*	0.039
E_ff_	0.11	0.11	0.004	0.002	0.006*	0.003

Seroconversors (Sc+), Non-seroconversors (Sc−).

*p-value <0.05 comparing Sc- versus Sc+

When analyzing the proportion of triple positive cells, CD38^+^DR^+^CCR5^+^, we found a similar distribution for T_INT_ (median of 0.015%), T_TM_ (median of 0.020%) and T_EM_ (median of 0.018%) subsets in Sc- group. Similar results were found for Sc+ group except for T_TM_ (median of 0.02%) which was more frequently (P<0.01) activated than T_EM_ (median of 0.01%). Again, T_CM_ was the less activated CD4^+^ T cell subset in both study groups. Comparing Sc+ versus Sc-, it was detected that more T_EM_ and T_Naive_ cells were activated in Sc- group (P = 0.02).

Finally, the expression of only CCR5 by CD4^+^ T cell subsets was higher for all subsets of T cell memory in Sc- group compared with Sc+ one. T_Naive_ cells of individuals from both groups had similar frequency of CCR5^+^ cells (Table 1).

Briefly, these data indicated that more differentiated memory T cell subsets showed higher immune activation than less differentiated T cells (T_Naive_ and T_CM_). T_INT_ subset expressing CD38 and DR was more frequent in individuals who seroconverted.

### Correlation between CD4^+^ T cell subsets and bactericidal antibodies against MenC

**Figure 3:**
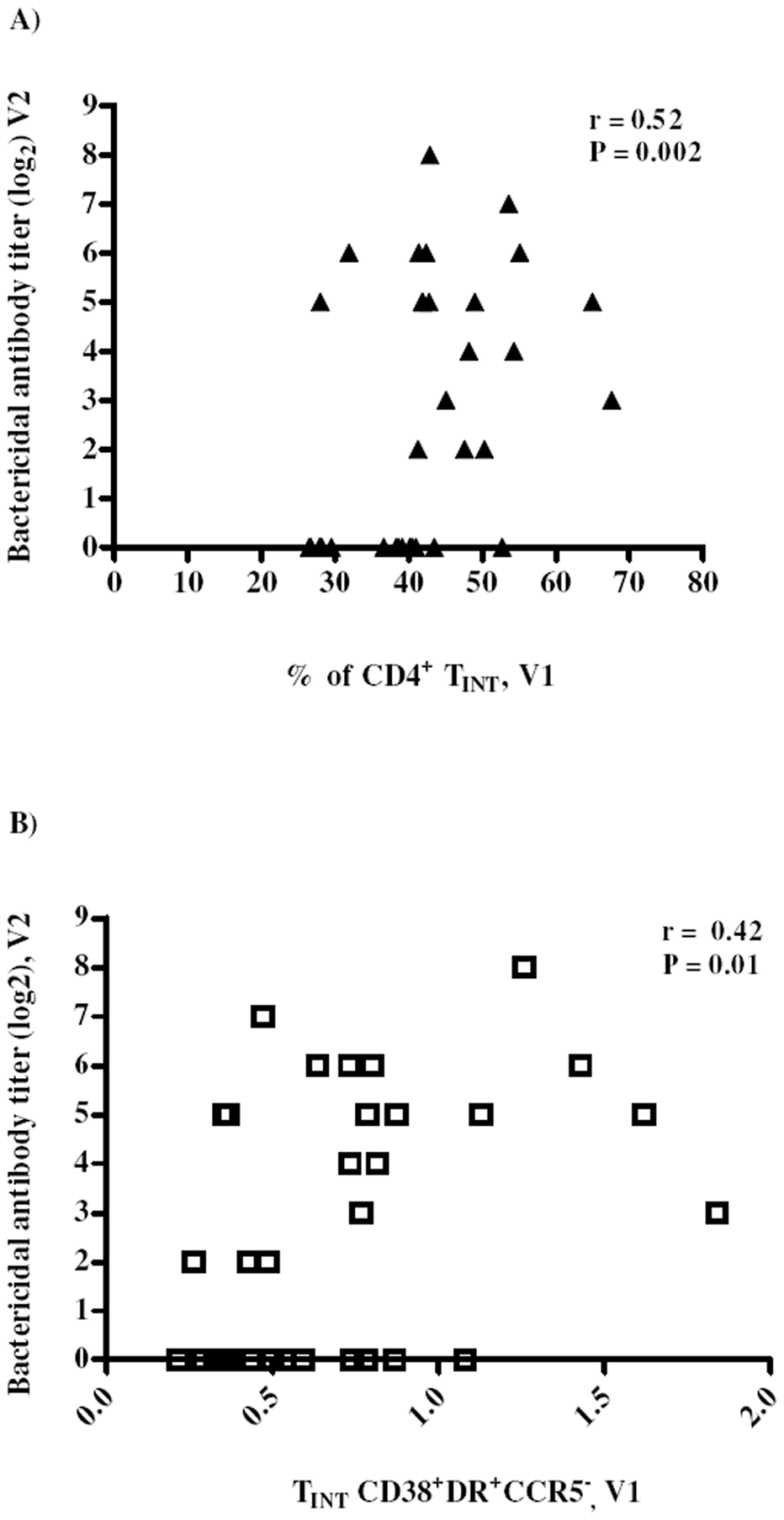
CD4^+^ T_INT_ memory subset correlates positively with bactericidal titers. (A) positive correlation between CD4^+^ T_INT_ frequency and (B) CD4^+^ T_INT_ CD38^+^DR^+^R5^−^ frequency before vaccination (V1) with post-vaccination bactericidal titers (V2). Correlations were analyzed using Sperman rank test.

After analyses of correlations of all CD4^+^ T cell subsets with bactericidal antibody titers (V2) we found a significant positive correlation (r = 0.52, P<0.01) only with T_INT_ subset in V1 (Fig. 3A). This finding may indicate that this CD4+ T cell subset is essential in mounting an effective vaccine response. A positive correlation (r = 0.42, P = 0.01) was still seen when analyzing activated (CD38^+^DR^+^) T_INT_ cells (V1) and bactericidal antibodies (Fig. 3B), indicating that HAART may be successfully limiting the excessive activation of these cells.

**Figure 4:**
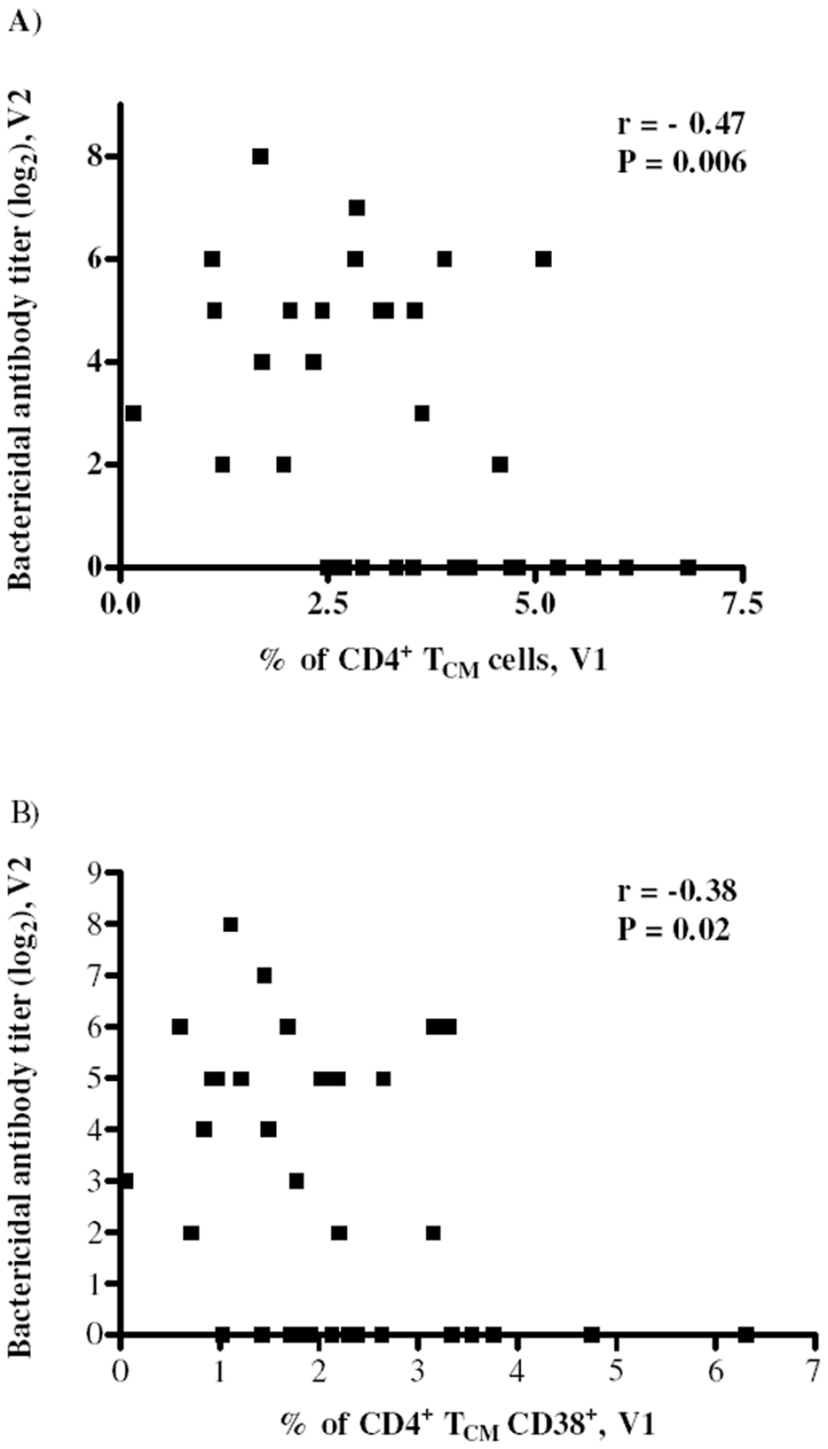
CD4^+^ T_CM_ memory subset correlates negatively with bactericidal antibody titers. Negative correlation between the frequency of CD4^+^ T_CM_ cells (A) and CD4^+^ T_CM_ cells expressing CD38 (B) before vaccination (V1) with post-vaccination bactericidal antibody titers (V2). Correlations were analyzed using Sperman rank test.

A negative correlation (r = −0.47, P<0.01) was detected between T_CM_ subset and bactericidal antibodies for both V1 (Fig. 4A) and V2 samples (data not shown). Fig. 4B shows that T_CM_ expressing only CD38 (median of 1.9%, data not shown) correlated negatively (r = −0.38, P = 0.02) with bactericidal antibodies. Similar result was found for V2 samples (median of 4.1%, data not shown). These data suggests that activation of a less differentiated cell, like T_CM_, have a significant effect in curbing CD4^+^ T cell function.

**Figure 5:**
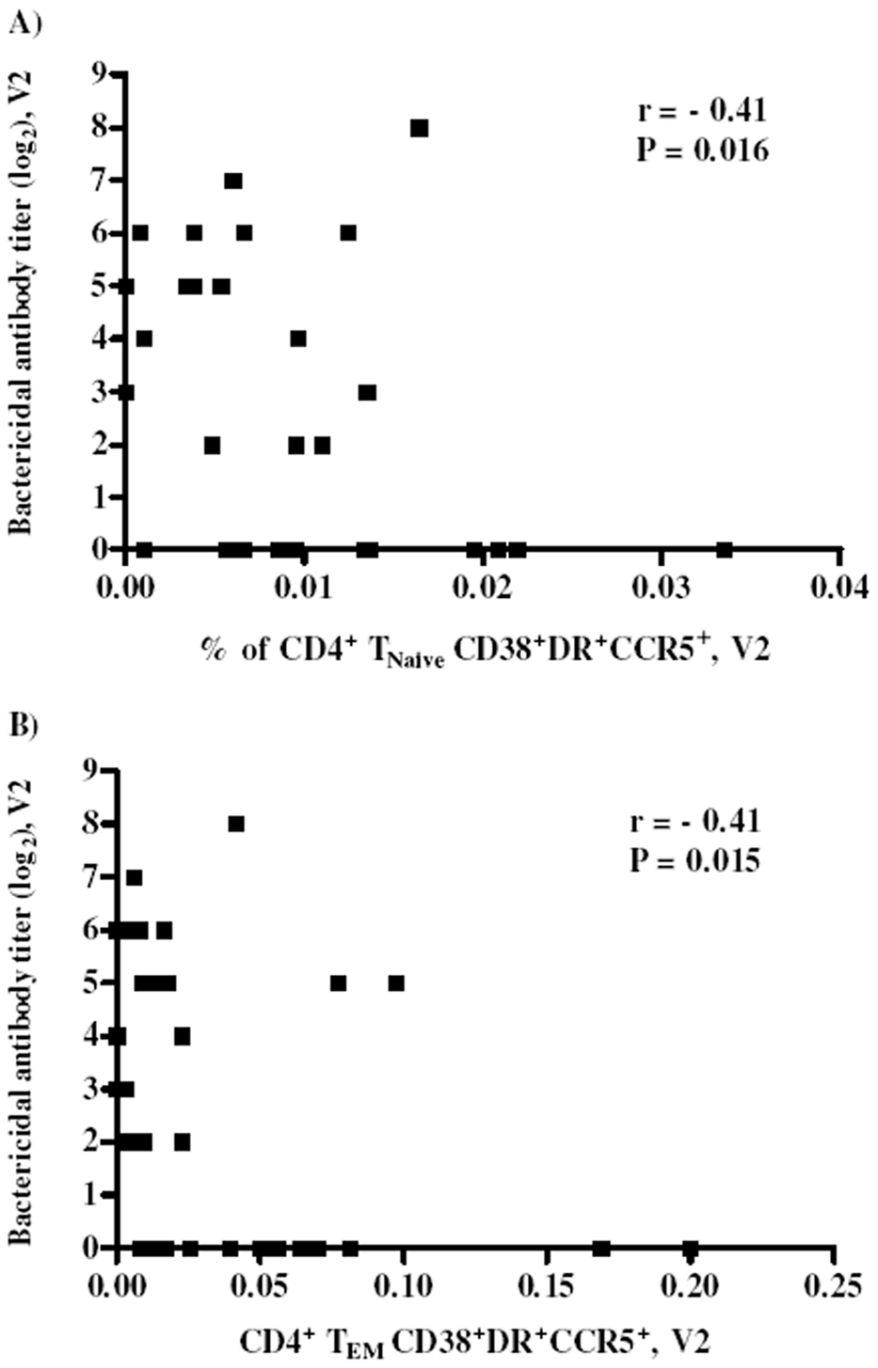
Triple positive CD4^+^ T cells negatively correlates with bactericidal antibody titers. CD4^+^ T_NAIVE_ (A) and T_EM_ (B) T cells with CD38^+^DR^+^CCR5^+^ phenotype correlates negatively with post-vacciantion bactericidal titers (V2). Correlations were analyzed using Sperman rank test.

Negative correlations with bactericidal antibodies were also detected for T_Naive_ and T_EM_ triple positive (CD38^+^DR^+^CCR5^+^) cells (Fig. 5 A and B) for both samples V2 and V1, suggesting that concomitant expression of CD38, HLA-DR and CCR5 have a significant impact in T cells, independent of the differentiation status of the cells.

## Discussion

It is widely accepted that quality, rather than quantity, of CD4^+^ and CD8^+^ T cell responses is more important in viral control and response to vaccines [2], [11], [12]. Phenotype and function of T cells are integrally linked, and reports have shown that stages of HIV-specific CD8^+^ T cell differentiation may be an important qualitative assessment [11], [12]. Similarly, identity of CD4^+^ T cell quality and its correlation with bactericidal antibody response may be important for a more rational use of vaccines against meningococci. Functional and usually high-affinity class-switched antibodies and memory B cells are products of the germinal center (GC). The CD4+ T cell help required for the development and maintenance of the GC is delivered by follicular Th cells (TFH), a CD4+ Thelper cell subset characterized by expression of Bcl-6 and secretion of IL-21 [16]. The cellular interactions that mediate differentiation of TFH and GC B cells remain an important area of investigation but a positive association between neutralizing antibodies and TFH cells after vaccination against H1N1 has been described [17]. The focus of this study was to investigate correlations between CD4^+^ T cell memory subsets and bactericidal antibody response after vaccination against MenC. Further investigations will be necessary to associate CD4^+^ T cell memory subsets and memory TFH cells.

In our study, HIV-infected children and adolescents who responded to vaccination against MenC had a predominance (about 44%) of CD4^+^ T_INT_ subset followed by T_TM_ subset (23 to 26%). As described by Burgers et al [11], T_INT_ cells are CD45RA^+^CD27^+^CCR7^−^, with few cells expressing CD57 (a marker of replicative senescence), and the proportion of cells expressing CD127 (the IL-7 receptor) are intermediate between T_CM_ and T_EM_. The use of CD27 is important to differentiate T_INT_ and T_Eff_ subset (CD45RA^+^CD27^−^CCR7^−^). Without CD27 marker we would have only a population of CD4^+^CD45RA^+^CCR7^−^ cells termed T_Eff_ or terminally differentiated effector cells (T_EMRA_), a subset highly heterogeneous in terms of CD27 and CD57 expression. Therefore the use of CD27 allowed us to distinguish between cells that may be functionally diverse. In fact, this study showed a positive correlation between the frequency of T_INT_ but not T_Eff_ subset and bactericidal antibody response to MenC.

Breton et al [10] using similar markers, except for CD27, did not select the T_INT_ subset and described similar frequencies of CD4^+^ T_CM_ and T_TM_ (about 17%), similar proportions of CD4^+^ T_EM_ and T_Eff_ (about 8%) and approximately 33% of CD4^+^ T_Naive_ cells in HIV uninfected individuals. In contrast, our data showed that the frequency of CD4^+^ T_CM_ subset was substantially low, peculiarly to responders compared to non-responders. Low number of CD4^+^ T_Naive_ cells was also detected in MenC vaccine responders. This shift toward a more differentiated T cell phenotype (T_INT_ and T_TM_) in responders may reflect a continuing priming of naïve T cells and their differentiation into a large population of intermediate and end-stage effectors cells.

Burgers et al [11] described similar frequencies (∼10%) of CD8^+^ T_TM_, T_INT_ and T_EM_ in HIV-exposed women. The lowest subpopulation being T_CM_ (less than 5%) and the highest (∼35%) the T_Eff_ subset of memory CD8^+^ T cells. Collectively, these data indicate that the frequency of T cell subsets varies according to the population studied and also to the definition of T cell subsets by flow cytometry. To date, we have not found any comprehensive report of CD4^+^ T memory subset frequencies in children and adolescents.

Importantly, in the HIV-infected children, CD4^+^ T_INT_ frequency was positively associated with bactericidal antibody response induced by vaccination. The positive correlation persisted despite the observation that T_INT_ subset activation (expression of CD38 and HLA-DR) was higher in responders. However, the frequency of CD38^+^DR^+^CD4^+^ T_INT_ cells was very low (median of 0.57%, minimum of 0.22% and maximum of 1.8%) which suggests that HAART was able to avoid a continuous activation of these cells. In contrast, CD4^+^ T_CM_ subset negatively correlated with bactericidal antibodies. The expression of the activation marker CD38 by this subset was also negatively associated with the antibody response. Therefore, our results indicate that the immune activation of CD4^+^ T cell memory subsets is heterogeneous and suggest that activation of less differentiated cells (e.g., T_CM_) have more functional impact than activation of more differentiated cells (e.g., T_INT_). Additional studies concerning to the cytokines secreted by these cells, as well as, the search for specific cell markers will be important to evaluate a possible interaction of CD4^+^ T cell memory subsets with germinal center B cells.

A similar analysis performed in aviremic-treated individuals [12] showed that HAART was able to restore the distribution of all memory CD4^+^ T cell subsets to the frequencies observed in healthy donors. However, HAART failed to restore normal CD8^+^ T cell subset frequencies, with persisting disequilibrium shown by lower numbers of T_CM_ and higher numbers of T_EM_ cell subsets compared with uninfected subjects.

Concluding, the lower frequency of CD4^+^ T cells at earlier stages of differentiation (T_Naive_ and T_CM_) in responders may indicate a better functional status of these cells and consequently a higher ability to proliferate and differentiate into effector cells after specific stimulus. The accumulation of T_INT_ subset in responders and its correlation with bactericidal antibody response indicate the importance to study the effector functions of specific T_INT_ cells. These issues should be addressed in future studies.
